# Esthetic Rehabilitation of a Discolored Maxillary Central Incisor With a High-Opacity Lithium Disilicate Crown

**DOI:** 10.7759/cureus.92150

**Published:** 2025-09-12

**Authors:** Linda Ayedi, Takoua Barhoumi, Zeineb Riahi, Dalenda Hadyaoui

**Affiliations:** 1 Fixed Prosthodontics, Dental Clinic of Monastir, University of Monastir, Monastir, TUN

**Keywords:** dental veneers, lithium disilicate crown, managment of dental trauma, maxillary central incisor, surgical crown lengthening, tooth discoloration

## Abstract

Intrinsic discoloration of maxillary central incisors is a common and challenging consequence of traumatic dental injuries, often resulting in compromised esthetics and complex restorative needs. This case report presents a detailed clinical and radiographic follow-up of the esthetic rehabilitation of a discolored maxillary central incisor in a 33-year-old female patient. Childhood trauma caused intrinsic discoloration and structural changes, including an immature apex and thin dentinal walls, complicating treatment. Initial examination revealed esthetic deficiencies and gingival inflammation associated with an old zirconia crown exhibiting a grayish cervical margin. Following removal of the failing prosthesis and crown lengthening surgery to reestablish biologic width and periodontal health, a high-opacity lithium disilicate crown was fabricated to effectively mask the deep discoloration from an underlying metal post. A lithium disilicate veneer was also placed on the adjacent lateral incisor to enhance symmetry and interdental spacing. Throughout treatment, meticulous multidisciplinary planning and adhesive protocols ensured functional and esthetic success. This report underscores that intrinsic discoloration secondary to trauma can present progressive restorative challenges requiring timely, customized surgical and prosthetic interventions to achieve lasting esthetic outcomes.

## Introduction

Traumatic injuries to permanent maxillary central incisors are relatively common during childhood, primarily due to their early eruption and exposed position within the dental arch [1,2]. A well-documented long-term consequence of such trauma is intrinsic discoloration, often secondary to pulpal necrosis or endodontic treatment [3]. Intracanal medicaments, obturation materials, and structural changes within the dentin can significantly alter the optical properties of the tooth, leading to progressive and noticeable discoloration over time [3].

When a single maxillary central incisor is affected, the esthetic impact is particularly significant due to its prominent location within the smile line. These teeth play a vital role not only in biting and speech but also in guiding jaw movements (anterior guidance) and shaping overall facial esthetics [4,5]. Matching the contralateral tooth in terms of color, shape, and surface texture poses a considerable restorative challenge, requiring both precision and interdisciplinary coordination. From the patient’s perspective, such defects can strongly influence self-confidence and quality of life.

Several restorative options exist to address discoloration, including direct composite restorations, porcelain-fused-to-metal crowns, and all-ceramic systems. However, composites may lack long-term stability in masking severe discoloration, and porcelain-fused-to-metal crowns often present esthetic limitations due to possible gingival shadowing. In contrast, lithium disilicate ceramics combine high esthetic potential, translucency control, and adequate strength, making them particularly well-suited for managing single discolored incisors while preserving tooth structure [4,6].

This case report aims to describe the multidisciplinary esthetic rehabilitation of a discolored maxillary left central incisor following childhood trauma, highlighting the decision-making process behind crown lengthening surgery, fiber post reconstruction, and the use of high-opacity lithium disilicate ceramics to achieve natural, symmetrical, and durable esthetic results.

## Case presentation

A 33-year-old female patient presented to the Fixed Prosthodontics Department of the Dental Clinic of Monastir, expressing dissatisfaction with her smile, stating: “This crown doesn’t look natural, and it makes me feel self-conscious when I speak or smile.” Her chief complaint was therefore both esthetic and psychological, with the unsatisfactory appearance of the existing zirconia crown on the maxillary left central incisor (tooth 21) negatively affecting her confidence in social interactions.

**Figure 1:**
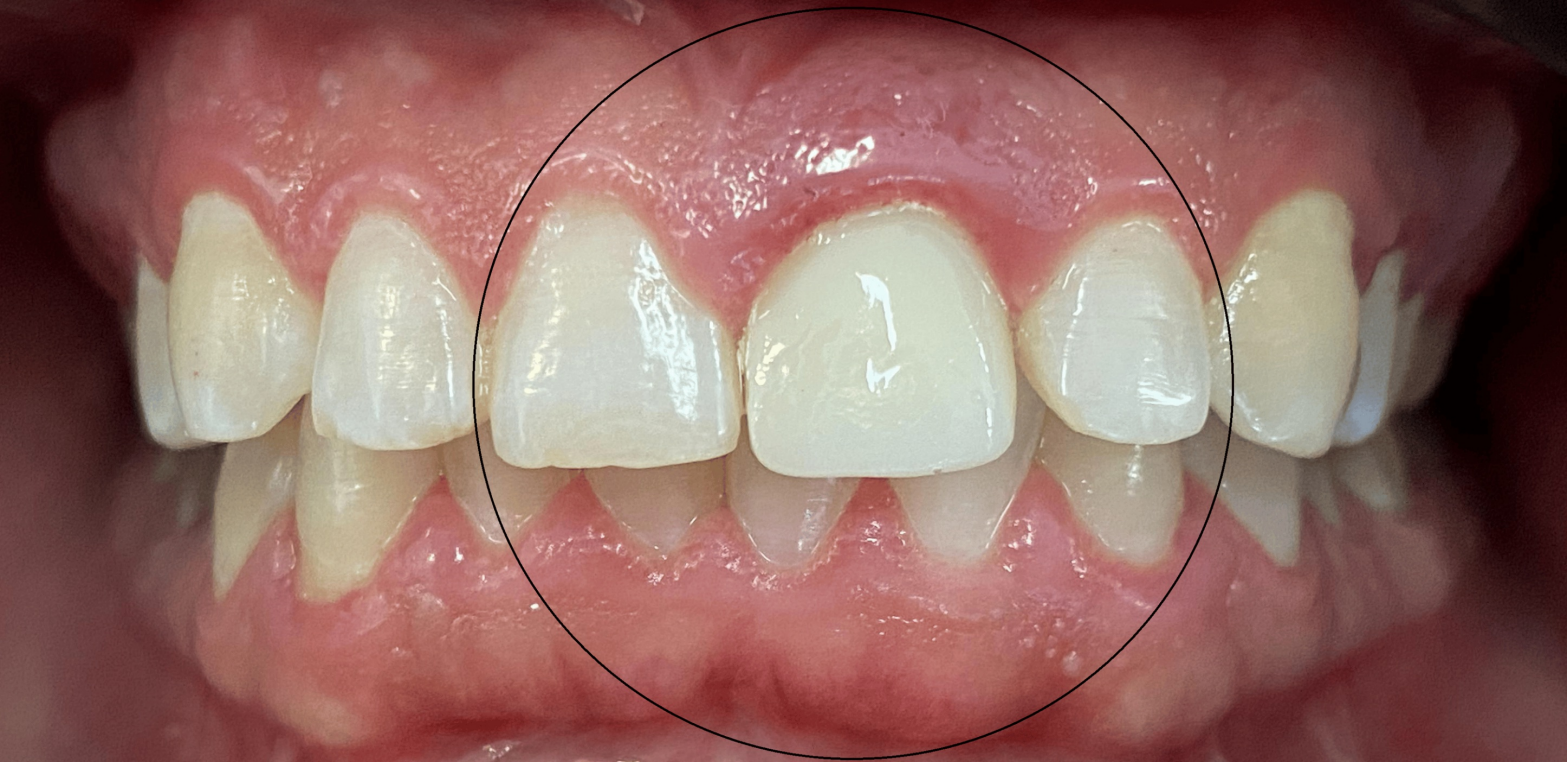
Intraoral view of the initial clinical presentation Intraoral frontal view showing the old full-coverage zirconia crown on tooth 21 with notable esthetic deficiencies: increased mesiodistal width, incisal edge misalignment, square-shaped morphology, and lack of color harmony with tooth 11. Tooth 22 also displays slight labial malposition contributing to the asymmetry.

Clinical examination revealed an old full-coverage zirconia crown on tooth 21 with several esthetic deficiencies compared to its contralateral tooth (11), including a wider mesiodistal dimension, incisal misalignment, a square-shaped morphology, and poor color integration. Tooth 22 was also slightly labially positioned (mildly displaced toward the lips). No functional disturbances, such as phonetic impairment or masticatory difficulty, were reported; however, the patient was primarily concerned with the esthetic imbalance in her anterior teeth (Figure 1).

Periodontal evaluation revealed acceptable oral hygiene but persistent gingival inflammation and a grayish discoloration around tooth 21. A discrepancy in the gingival zenith (the highest point of the gingival margin, which influences smile harmony) between teeth 21 and 11 was noted. Pocket probing with a periodontal probe measured 1.5 mm, and bitewing radiography confirmed a violation of the biologic width (the natural 2.04 mm space required between the alveolar bone crest and gingival margin to maintain periodontal health). This finding indicated the need for crown lengthening surgery to restore gingival balance and ensure long-term stability.

**Figure 2:**
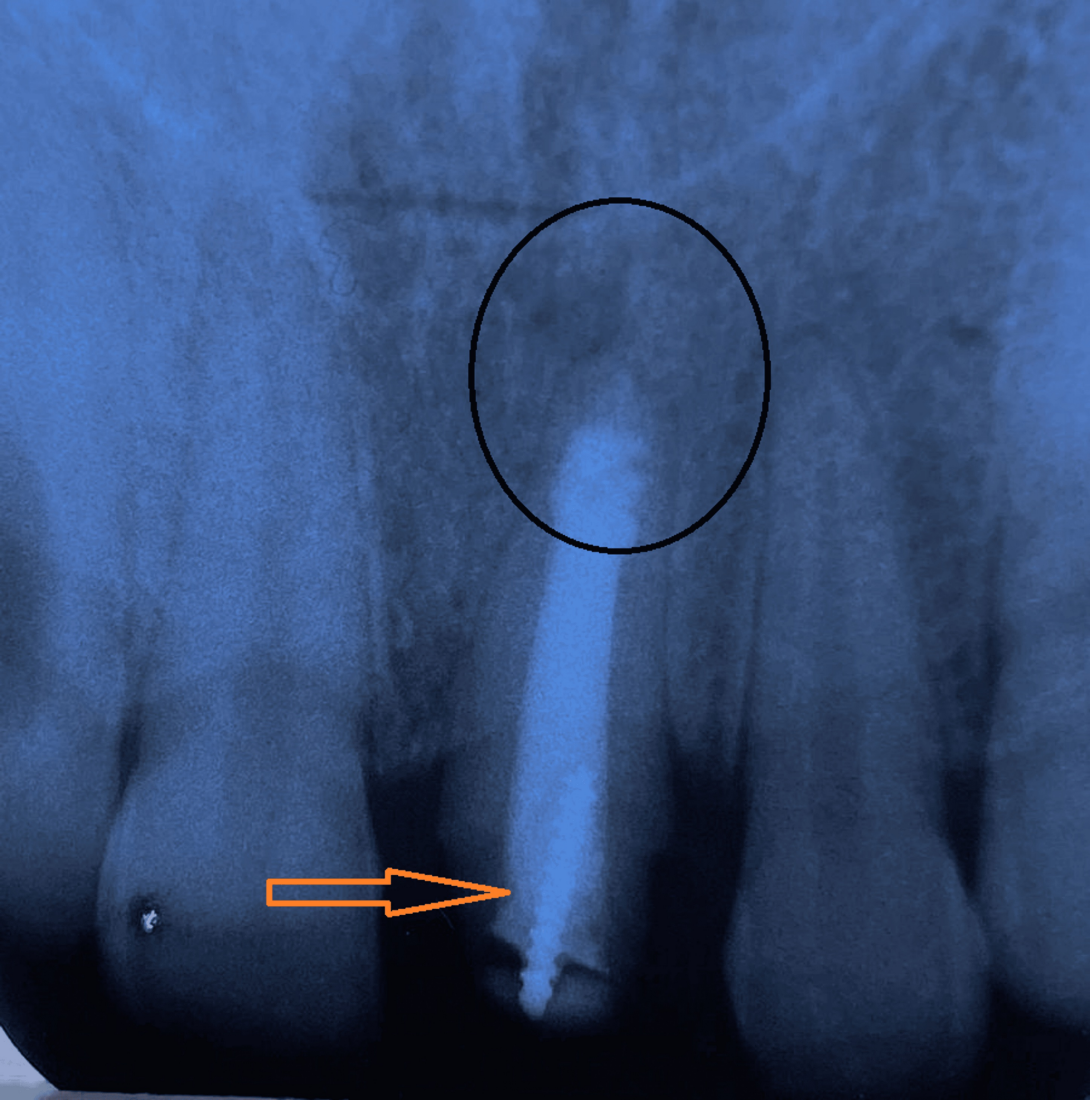
Retro alveolar X-ray of tooth 21 Radiographic examination reveals an immature tooth 21 with an open apex and thin dentinal walls, consistent with previous traumatic injury. Endodontic treatment appears complete, with no signs of periapical pathology. A short metallic post is embedded in the obturation material.

Radiographically, tooth 21 showed signs of an immature apex and thin dentinal walls consistent with prior trauma. Endodontic treatment had already been completed, with no evidence of periapical pathology or root fractures. A short (3 mm) metallic post was incorporated within the obturation material (Figure 2).

A comprehensive pre-prosthetic assessment was initiated, including clinical photography, smile analysis, shade selection, and a diagnostic wax-up. This evaluation revealed the need for a veneer on tooth 22 to correct its minor labial malposition, optimize interdental spacing, and enable the fabrication of a proportionally matched prosthesis on tooth 21. The analysis also highlighted the necessity to harmonize the gingival zeniths of the anterior teeth by aligning the cervical margins, thereby improving overall gingival symmetry. Based on these findings, a treatment plan was established: a monolithic lithium disilicate crown for tooth 21 and a lithium disilicate veneer for tooth 22.

**Figure 3:**
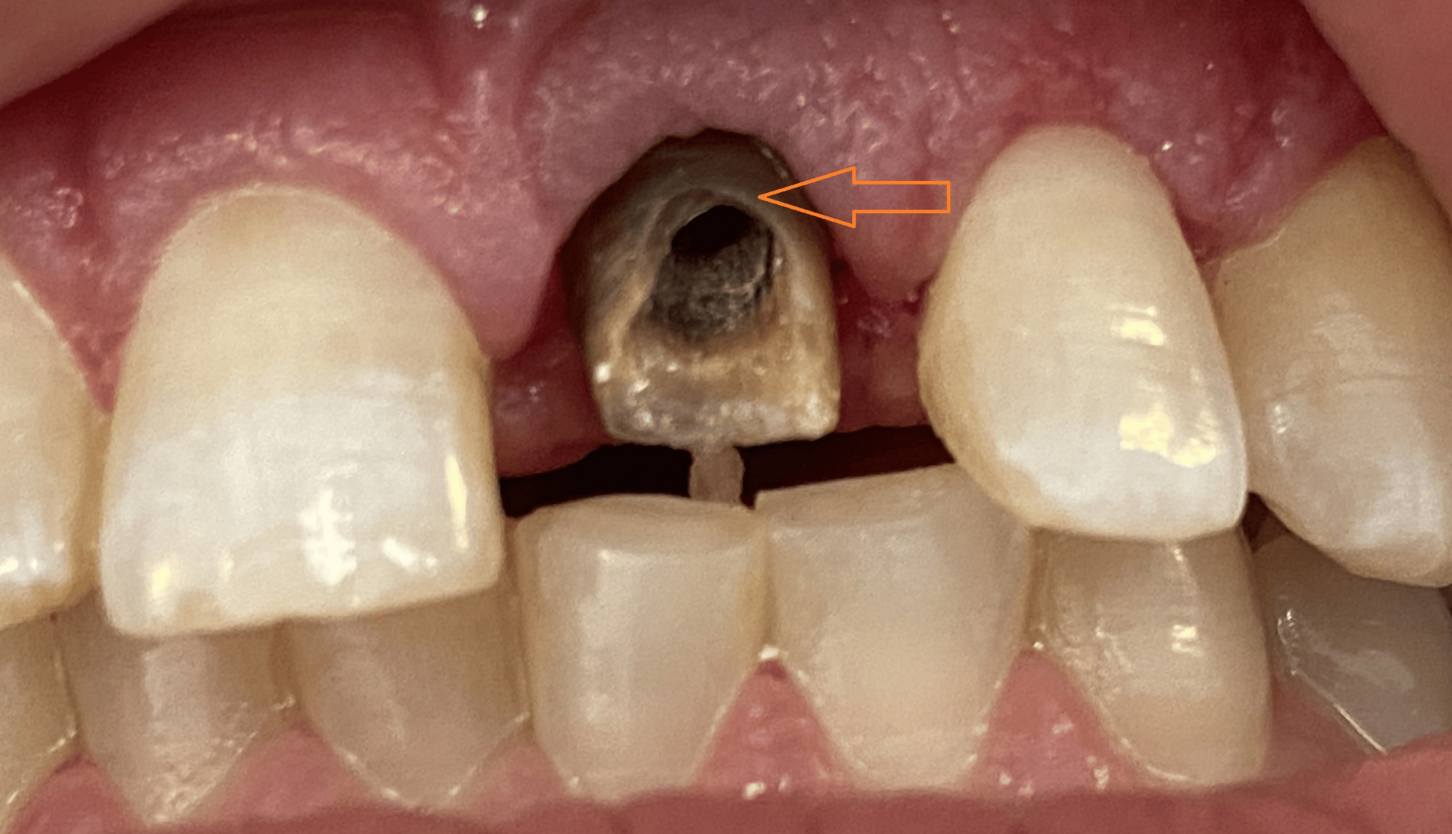
Removal of the prefabricated metallic post revealing subgingival discoloration and structural compromise in tooth 21 The image shows the maxillary anterior teeth of a patient with the upper left central incisor (tooth 21) exposed. The crown has been removed, revealing a severely discolored dentin core with a visible prefabricated metal post. The remaining tooth structure is notably compromised. The buccal surface exhibits subgingival exposure, while the lingual and proximal margins appear supragingival. Adjacent teeth seem intact, although slight malalignment is visible.

Upon removal of the old crown, a defective resin restoration retained by a prefabricated metal post was exposed. The assembly was carefully removed with an ultrasonic tip to retrieve the post without compromising the remaining structure. The underlying dentin on tooth 21 was severely discolored with the shade of ND9 (IPS Natural die Material d’ IVOCLAR Vivadent), with subgingival exposure on the buccal surface and supragingival margins on the lingual and proximal sides (Figure 3). 

**Figure 4:**
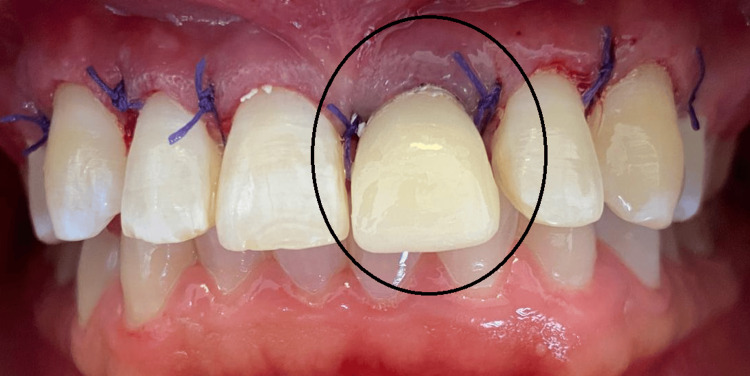
Post-surgical view following crown lengthening and provisionalization Crown lengthening surgery was performed from canine to canine to correct altered passive eruption, resulting in a 2 mm increase in the clinical crown height of the anterior teeth. An osteotomy was selectively carried out on tooth 21 to reestablish biologic width. A bis-acrylic provisional restoration (shade A2) was temporarily cemented to guide soft tissue healing and support the gingival contour.

To correct the gingival asymmetry through cervical margin alignment, a gingivectomy (reshaping of gum margins) was performed from canine to canine. In addition, crown lengthening with osteotomy (minor bone recontouring to reestablish the biologic width) was selectively carried out on tooth 21 to ensure periodontal health. A bis-acrylic provisional crown (shade A2) was fabricated and temporarily cemented with eugenol-free cement to guide soft tissue healing and promote proper gingival architecture (Figure 4).

After healing, the remaining tooth structure was re-evaluated. Gingival probing depths had increased from 1.5 mm preoperatively to 3 mm, confirming the successful reestablishment of the biologic width. A fiber post (size 3, tapered) was then placed in tooth 21 using dual-cure self-adhesive resin cement to reinforce the weakened root. Full-coverage crown preparation was completed for tooth 21, while a minimally invasive veneer preparation was carried out on tooth 22.

**Figure 5:**
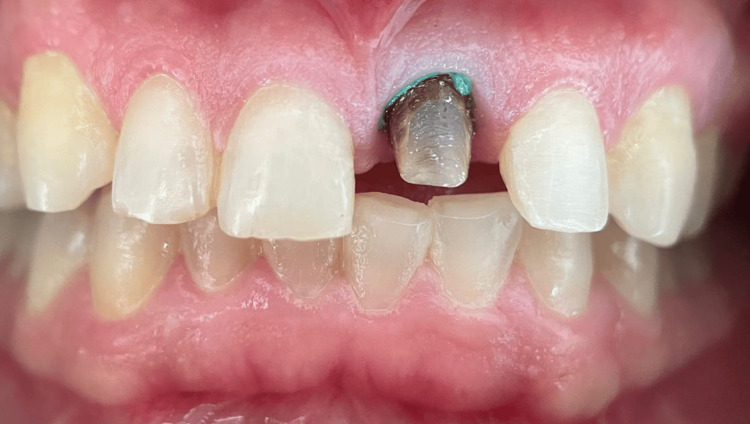
Tooth preparation on teeth 21 and 22 Tooth 21 was prepared for a full-coverage crown, while a minimally invasive veneer preparation was performed on tooth 22. The preparations were designed to ensure optimal esthetic integration, preservation of tooth structure, and proper spatial distribution for final ceramic restorations.

Gingival retraction was achieved using retraction cords, and a double-mix impression technique with addition silicone (Elite HD, Zhermack) captured the tooth morphology and surrounding soft tissue architecture. Final shade selection was performed using the VITA 3D-MASTER guide, with 2M2 selected (Figure 5).

For tooth 21, a 1.2 mm high-opacity (HO) lithium disilicate coping (IPS e.max Press) was fabricated. The final crown and veneer were both pressed from medium translucency (MT) IPS e.max blocks.

During the try-in stage, fit, marginal integrity, symmetry, contact points, and esthetic integration were evaluated and approved by the patient. After selecting the appropriate cement shade with a water-soluble try-in paste, all ceramic surfaces were cleaned in 95% alcohol using ultrasonic agitation, etched with 4% hydrofluoric acid (30 seconds), primed (Bisco), and silanized (Bis-Silane A and B).

**Figure 6:**
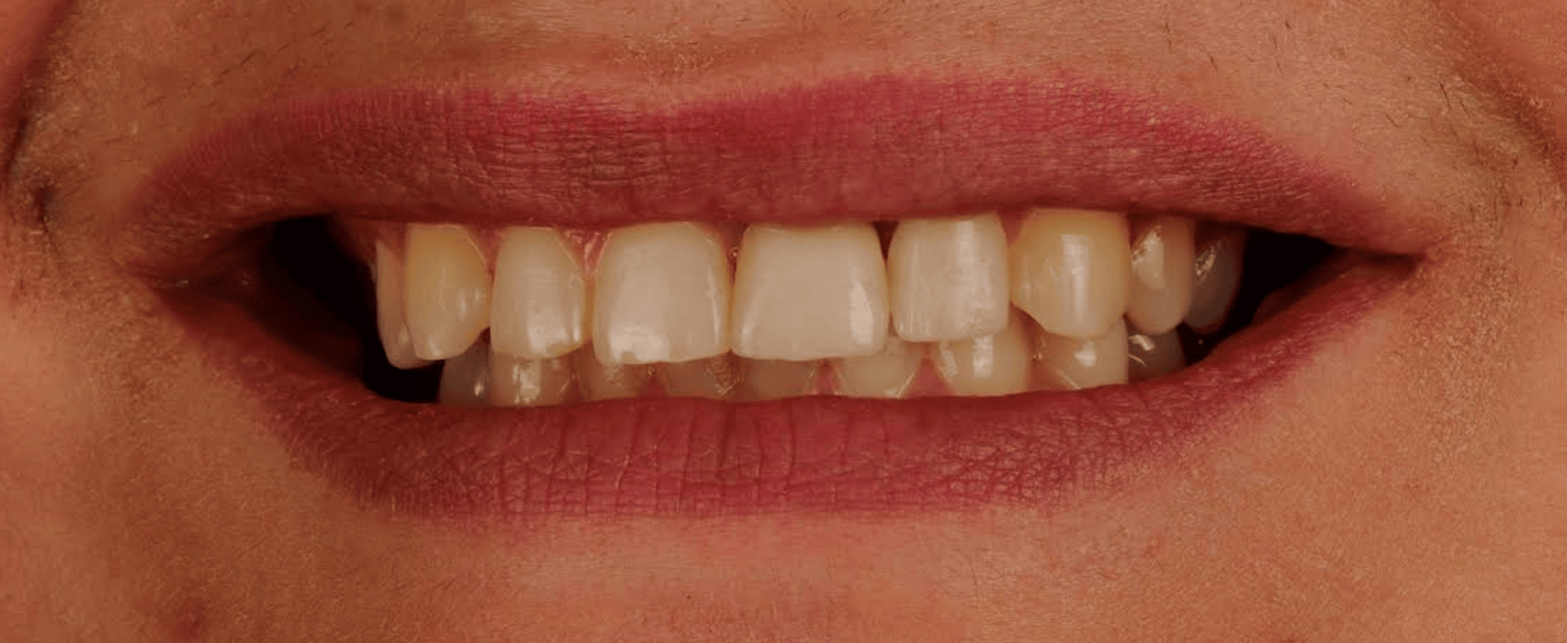
Final extraoral view after cementation of the restorations The lithium disilicate crown on tooth 21 and the veneer on tooth 22 were bonded using a light-cure resin cement. Finishing and polishing were achieved using a dedicated kit to refine occlusal anatomy and ensure smooth, well-integrated restorative margins. The final result demonstrates harmonious color match, symmetry, and natural esthetics.

Tooth surfaces were cleaned with non-fluoridated prophy paste, etched with 37% phosphoric acid (30 seconds for enamel, 15 seconds for dentin), and treated with an adhesive primer (All-Bond 3, Bisco). The bonding resin (Choice 2, Bisco) was applied to the intaglio surfaces, and restorations were seated. Initial light polymerization (5 seconds) facilitated excess cement removal, followed by complete curing (40 seconds per surface). Final finishing and polishing were done with a dedicated kit to refine occlusion and smooth margins (Figure 6).

**Figure 7:**
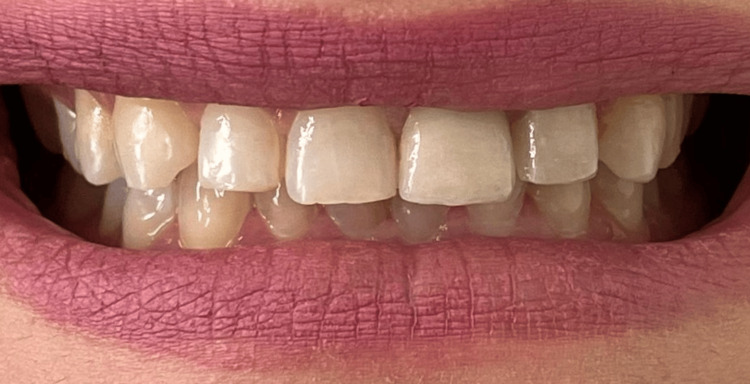
Six-month follow-up after esthetic and functional restoration

At the six-month recall, the patient reported high satisfaction with the esthetic and functional outcome. Clinical examination revealed stable soft tissues with harmonious gingival contours and no signs of inflammation. Probing depths remained within normal limits (approximately 3 mm), confirming the long-term stability of the surgically reestablished biologic width. Both the lithium disilicate crown on tooth 21 and the veneer on tooth 22 demonstrated excellent marginal adaptation, color stability, and surface integrity, with no evidence of chipping, debonding, or secondary caries. The restorations maintained a natural integration with the adjacent teeth, and phonetic and functional parameters were fully preserved (Figure 7).

## Discussion

This case highlights the clinical complexity of managing intrinsic discoloration in a traumatized maxillary central incisor with additional structural and periodontal complications. The etiology of the discoloration was primarily related to the presence of a prefabricated metallic post, which had corroded and contributed to deep staining of the surrounding dentin, an observation consistent with findings in the literature, where metallic posts have been linked to persistent discoloration and esthetic failure [7].

The decision to avoid internal bleaching was based on the severity and depth of the discoloration. Although bleaching is often considered a minimally invasive method for non-vital tooth discoloration [7], it was deemed ineffective in this case due to the dark metallic staining and severely compromised dentin walls. Instead, a high-opacity (HO) lithium disilicate crown (IPS e.max HO) was selected for its superior shade-masking ability. This choice is supported by multiple studies showing that HO E.max copings with a thickness of 0.5-0.6 mm effectively mask dark backgrounds and outperform white zirconia copings (Lava™ Zirconia) of similar thickness [8,9].

The clinical success of ceramic restorations in discolored substrates depends largely on the selection of materials with appropriate optical properties. The HO E.max coping provided an effective barrier against the underlying discoloration, while the superstructure pressed from a medium-translucency E.max ingot allowed for lifelike esthetics. Literature supports this approach, emphasizing the balance between opacity for masking and translucency for natural appearance [8,10].

However, the presence of a grayish cervical discoloration in the gingival tissues, likely due to biologic width violation and subgingival placement of the old restoration, could not be corrected by the ceramic crown alone. Surgical crown lengthening was indicated to reestablish biologic width and eliminate subgingival inflammation. As established by Gargiulo et al., maintaining a biologic width of 2.5-3 mm is essential to ensure periodontal health and long-term restoration success [11,12]. In this case, flap elevation and osseous recontouring around tooth 21 were performed to correct altered passive eruption, improve gingival symmetry, and resolve the cervical shadowing, ultimately restoring healthy architecture.

The second clinical consideration was achieving symmetry and harmony in the anterior maxillary segment, especially between teeth 11 and 21. Tooth 21 presented a square morphology, greater mesiodistal width, and incisal misalignment, while tooth 22 was slightly labially positioned. To optimize esthetics and redistribute interdental spacing, a ceramic veneer was placed on tooth 22. This allowed us to correct minor alignment issues and achieve better balance and proportion across the smile line. Conservative ceramic veneers, particularly micro-veneers with a thickness of 0.3 to 0.7 mm, are effective in such cases for shape modification and space management while preserving enamel integrity [13,14].

From a clinical standpoint, the treatment plan was driven by three main factors: the need to mask deep intrinsic discoloration from a metallic post, the presence of periodontal compromise due to biologic width violation, and the goal of restoring anterior esthetics and symmetry. The combination of crown lengthening, fiber post reconstruction, a high-opacity lithium disilicate crown, and a ceramic veneer provided a conservative yet comprehensive solution tailored to the patient’s functional and esthetic needs. Patient satisfaction was consistent with findings from Jantea et al., who reported a 93% satisfaction rate in cases involving combined endodontic and prosthetic management of dental dyschromia [15], underscoring the importance of a multidisciplinary approach and thoughtful material selection.

Despite the successful outcome, this case presented several challenges. One limitation was the difficulty in correcting the grayish cervical discoloration, which required additional surgical intervention. The use of a high-opacity lithium disilicate crown was effective, but it also required precise planning to ensure that the material properties sufficiently masked the deep discoloration. Moreover, while the treatment successfully restored esthetics and function, it is important to note that the long-term stability of the restorations, particularly in terms of color and gingival health, will require continuous monitoring. The presence of a subgingival restoration in the past highlighted the importance of maintaining a biologic width and avoiding subsequent periodontal complications.

This long-term follow-up reinforces the importance of a multidisciplinary approach in managing complex anterior tooth discoloration, addressing both esthetic concerns and functional needs. Cases involving combined endodontic and prosthetic management of dental dyschromia show a high patient satisfaction rate, underlining the significance of tailored, comprehensive treatment plans to meet the psychological and esthetic expectations of patients.

## Conclusions

Managing esthetic challenges in dyschromic maxillary anterior teeth requires a thoughtful, interdisciplinary approach. This case illustrates how surgical, structural, and restorative considerations, such as biologic width reestablishment, correction of gingival asymmetry, and the use of high-opacity lithium disilicate ceramics, can be integrated to achieve predictable, esthetically pleasing results.

Digital tools and minimally invasive techniques further enhanced precision and soft tissue outcomes. This case underscores the importance of tailored treatment planning, material selection, and collaboration across specialties in addressing complex anterior esthetic demands.
